# A biodegradable in-situ polymer precipitation delivery system for sustained delivery of a novel chimeric natriuretic peptide CD-NP in an experimental model of myocardial infarction

**DOI:** 10.1186/2050-6511-14-S1-P38

**Published:** 2013-08-29

**Authors:** Soo Ghim Lim, S Jeson Sangaralingham, Syed Ameenuddin, Fernando L Martin, Xu Wen Ng, Ying Ying Huang, Subbu S Venkatraman, John C Burnett, Horng H Chen

**Affiliations:** 1 School of Materials Science and Engineering, Nanyang Technological University, Singapore; 2 Cardiorenal Research Laboratory, Division of Cardiovascular Diseases, Mayo Clinic, Rochester, Minnesota, USA

## Background

CD-NP (Cenderitide) is the only chimeric natriuretic peptide that acts on both the A and B natriuretic peptide receptors (NPR). This differential mechanism of action reduces the arterial vasodilating properties, while retaining the cardiac unloading and aldosterone inhibiting actions of the NPR-A receptor in addition to the direct anti-remodeling actions of the NPR-B receptor. Our objective is to determine the effects of 3 weeks of sustained delivery of CD-NP via a single subcutaneous (SQ) injection of a fully biodegradable in-situ polymer precipitation delivery system (Gel system) in a model of experimental myocardial infarction (MI).

## Materials and methods

The CD-NP Gel system consists of 0.45% percentage weight/weight (w/w) CD-NP formulated with 40% Poly (lactic-co-glycolic acid) in 39.55% w/w N-methyl-2-pyrrolidinone and 20% w/w triacetin. Coronary (LAD) ligation rat (Wistar Male, 250-300g) model of MI was used: Treatment group (CD-NP Gel, n=10) and Vehicle group (Gel only, n=8). CD-NP gel or gel alone was administered via a single SQ injection with induction of MI. The delivery efficacy of the CD-NP Gel system, cardiac function and structure, and cGMP were assessed 3-weeks post MI.

## Results

Plasma and urinary evaluations demonstrated a significant elevation for both CD-NP and the second messenger cGMP in the treatment group at 3 weeks as compared to vehicle group: plasma CD-NP 5,000 ± 505 pg/ml vs 120 ± 6.2 pg/ml; plasma cGMP 170 ± 21.4 pmol/ml vs 112 ± 8.0 pmol/ml respectively. This confirms the CD-NP gel system’s efficacy and the biological activity of released peptide. Both mean arterial blood pressure and ejection fraction at 3-weeks were found to be significantly higher in treated group as compared to vehicle group, thus suggesting improvements in overall cardiac function. Heart/body weight ratio in treated group was found to be significantly lower as compared to vehicle group. Left ventricular fibrosis and collagen deposition was also significantly reduced in treated rats. (p<0.05 comparing Treatment group vs Vehicle group for the above mentioned parameters).

## Conclusion

This study demonstrates the efficacy and feasibility of a novel chronic peptide polymer gel delivery system for the dual NPR-A/B chimeric peptide CD-NP. Specifically, a one time SQ injection of CD-NP Gel resulted in 3 weeks sustained delivery of the functional peptide with favorable cGMP activating actions and improved cardiac remodeling in this model of experiment MI. Further studies are warranted to translate the current study to human MI.

